# 3-Benzyl-5-methyl-1,2-benzoxazole 2-oxide

**DOI:** 10.1107/S160053681203838X

**Published:** 2012-09-19

**Authors:** G. Anuradha, Vasuki Gopalsamy, A. Veera Reddy, G. Laxminarasimhulu

**Affiliations:** a Department of Physics, Kunthavai Naachiar Government Arts College (w) (Autonomous), Thanjavur-7, India; b R & D Department, Suven Life Sciences Ltd, Hyderabad-55, Andhra Pradesh, India

## Abstract

In the title compound, C_15_H_13_NO_2_, the isoxazole unit and the attached benzene ring are almost coplanar, making a dihedral angle of 1.42 (8)°. The benzyl ring is inclined to the isoxazole ring by 74.19 (8)° and is in a +*sc* conformation with respect to the benzisoxazole unit. In the crystal, C—H⋯O hydrogen bonds link the mol­ecules, forming zigzag chains propagating along the *b* axis. There are also π–π inter­actions present involving the isoxazole and benzyl rings [centroid–centroid distance = 3.5209 (10) Å], and C—H⋯π inter­actions involving the benzene ring of the benzoisoxazole unit and the methyl­ene bridging group.

## Related literature

For the anti-epileptic, anti­spasmodic and anti­fungal properties of benzoxazole derivatives, see: Jian *et al.* (2007 ▶). For their anti­tuberculer activity, see: Vinšová *et al.* (2007 ▶). For other biological activties of isoxazoles and benzisoxazole derivatives, see: Veera Reddy *et al.* (2011 ▶). For details of the synthesis, see: Veera Reddy *et al.* (2011 ▶). For the related structure 5-chloro-3-methyl-1,2-benzisoxazole-2-oxide, see: Ghari & Viterbo (1982 ▶).
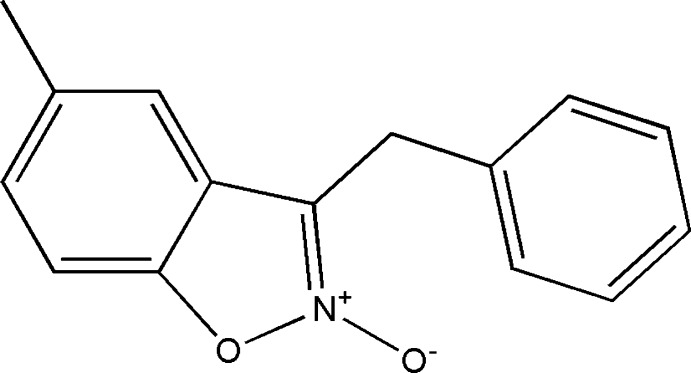


## Experimental

### 

#### Crystal data

C_15_H_13_NO_2_
*M* *_r_* = 239.26
Monoclinic, 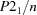

*a* = 6.4527 (2) Å
*b* = 11.2213 (4) Å
*c* = 16.9371 (7) Å
β = 100.002 (2)°
*V* = 1207.74 (8) Å^3^
*Z* = 4
Mo *K*α radiation
μ = 0.09 mm^−1^
*T* = 293 K
0.30 × 0.20 × 0.20 mm

#### Data collection

Bruker Kappa APEXII CCD diffractometer
Absorption correction: multi-scan (*SADABS*; Bruker, 1999 ▶) *T*_min_ = 0.974, *T*_max_ = 0.983
13491 measured reflections
3512 independent reflections
2113 reflections with *I* > 2σ(*I*)
*R*_int_ = 0.026

#### Refinement

*R*[*F* ^2^ > 2σ(*F* ^2^)] = 0.050
*wR*(*F* ^2^) = 0.172
*S* = 1.06
3512 reflections
163 parameters
H-atom parameters constrained
Δρ_max_ = 0.26 e Å^−3^
Δρ_min_ = −0.20 e Å^−3^

### 

Data collection: *APEX2* (Bruker, 2004 ▶); cell refinement: *SAINT-Plus* (Bruker, 2004 ▶); data reduction: *SAINT-Plus*; program(s) used to solve structure: *SHELXS97* (Sheldrick, 2008 ▶); program(s) used to refine structure: *SHELXL97* (Sheldrick, 2008 ▶); molecular graphics: *ORTEP-3 for Windows* (Farrugia, 1997 ▶) and *PLATON* (Spek, 2009 ▶); software used to prepare material for publication: *WinGX* publication routines (Farrugia, 1999 ▶).

## Supplementary Material

Crystal structure: contains datablock(s) I, global. DOI: 10.1107/S160053681203838X/su2493sup1.cif

Structure factors: contains datablock(s) I. DOI: 10.1107/S160053681203838X/su2493Isup2.hkl

Supplementary material file. DOI: 10.1107/S160053681203838X/su2493Isup3.cml

Additional supplementary materials:  crystallographic information; 3D view; checkCIF report

## Figures and Tables

**Table 1:** Hydrogen-bond geometry (Å, °) *Cg*2 is the centroid of the C2–C7 ring.

*D*—H⋯*A*	*D*—H	H⋯*A*	*D*⋯*A*	*D*—H⋯*A*
C5—H5⋯O2^i^	0.93	2.49	3.154 (2)	128
C8—H8*B*⋯*Cg*2^ii^	0.97	3.00	3.6800 (16)	129

Symmetry codes: (i) 
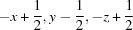
; (ii) 
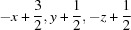
.

**Fig. 1.**
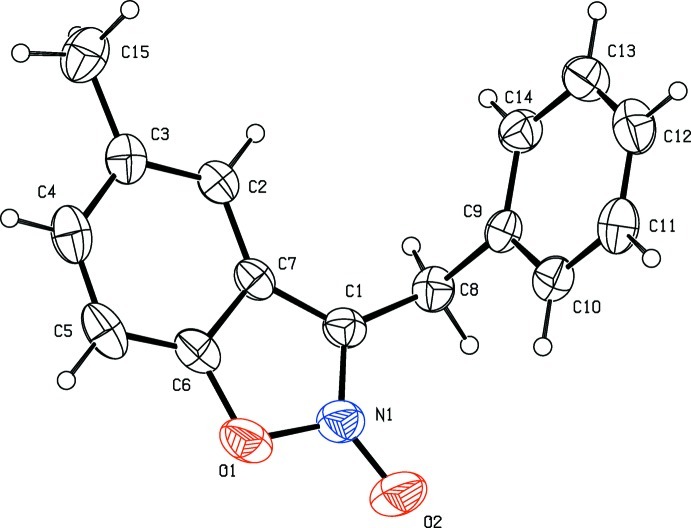
The molecular structure of the title molecule, with the atom numbering. Displacement ellipsoids are drawn at the 50% probability level.

**Fig. 2.**
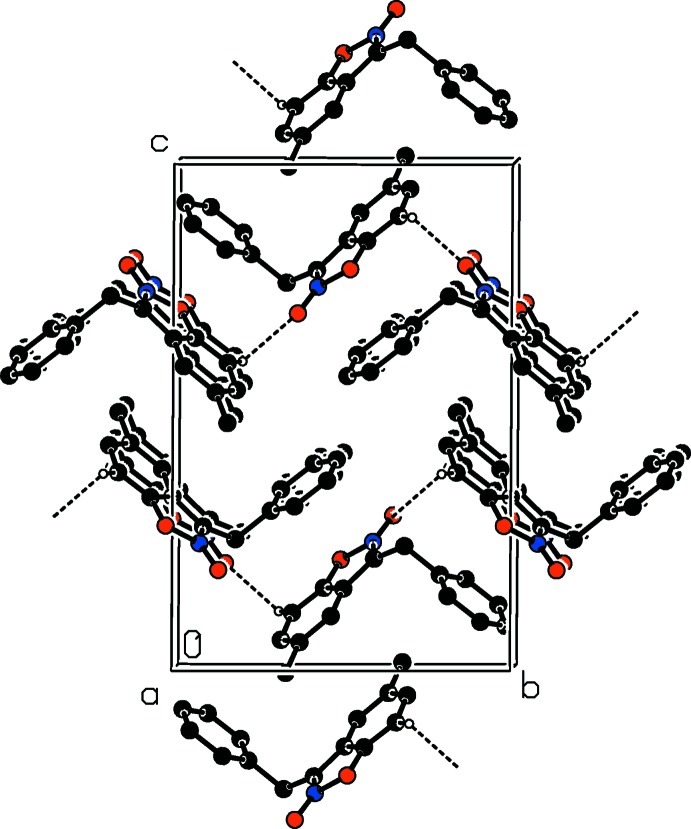
A view along the a axis of the crystal packing of the title compound. The intermolecular C—H···O hydrogen bonds are shown as dashed lines (see Table 1 for details; H atoms not involved in these interactions have been omitted for clarity).

## supplementary crystallographic information

### Comment

Isoxazoles and benzisoxazoles are important classes of nitrogen-oxygen containing heterocycles. They have extensive biological applications and are useful intermediates in medicinal chemistry (Veera Reddy *et al.*, 2011). The benzoxazole skeleton is an essential structural unit of several antibacterial, anticancer and anti-HIV-1 agents. The antituberculotic activity of several benzoxazole derivatives have been reported (Vinšová *et al.*, 2007). Some benzoxazoles exhibit high fluorescence and are used as optical whitening agents, photoluminesents and active components in dye lasers. Benzoxazole derivatives show antiepileptic, antispasmodic and antifungal properties (Jian *et al.*, 2007). 3-substituted 1,2-benzisoxazole derivatives are emerging as potential antipsychotic compounds, antiseizure agents and are also used to block the repetitive firing of voltage-sensitive sodium channels and so reduce voltage-sensitive T-type calcium currents (Veera Reddy *et al.*, 2011).

The molecular structure of the title functionalized 1,2-benzisoxazole compound is illustrated in Fig. 1. It contains three planar rings, namely, a methyl substituted benzene ring A = C2—C7, an isoxazole ring B =C1/C7/C6/O1/N1 and the benzyl ring C = C9—C14. The dihedral angles between rings A/B and B/C are 1.42 (8)° and 74.19 (8)°, respectively.

The bond lengths and angles in the title compound are in good agreement with the expected values and are comparable with the corresponding values reported for 5-chloro-3-methyl-1,2-benzisoxazole-2-oxide (Ghari & Viterbo, 1982).

In the crystal, molecules are linked *via* C—H···O hydrogen bonds leading to the formation of zigzag chains propagating along the *a* axis direction (Tabel 1 and Fig. 2). Molecules are also linked *via* C—H···π (Table 1) and π···π interactions. The latter involve the isoxazole (B = *Cg*1) and benzyl rings (C = *Cg*3) [*Cg*1···*Cg*3^i^ = 3.5209 (10) Å; symmetry code: (i) -*x* + 1.5, *y* - 1/2, -*z* + 1/2].

### Experimental

The compound was synthesized by the published method (Veera Reddy *et al.*, 2011)

### Refinement

All the H atoms were positioned geometrically and treated as riding atoms: C—H = 0.93, 0.96 and 0.97 Å for CH, CH_3_ and CH_2_ H atoms, respectively, with *U*_iso_(H) = k × *U*_eq_(C), where k = 1.5 for CH_3_ H atoms and = 1.2 for other H atoms.

### Crystal data

**Table:**

C_15_H_13_NO_2_	*F*(000) = 504
*M**_r_* = 239.26	*D*_x_ = 1.316 Mg m^−^^3^
Monoclinic, *P*2_1_/*n*	Mo *K*α radiation, λ = 0.71073 Å
Hall symbol: -P 2yn	Cell parameters from 13955 reflections
*a* = 6.4527 (2) Å	θ = 1.2–30.1°
*b* = 11.2213 (4) Å	µ = 0.09 mm^−^^1^
*c* = 16.9371 (7) Å	*T* = 293 K
β = 100.002 (2)°	Block, colourless
*V* = 1207.74 (8) Å^3^	0.30 × 0.20 × 0.20 mm
*Z* = 4	

### Data collection

**Table:**

Bruker Kappa APEXII CCD diffractometer	3512 independent reflections
Radiation source: fine-focus sealed tube	2113 reflections with *I* > 2σ(*I*)
Graphite monochromator	*R*_int_ = 0.026
ω and φ scan	θ_max_ = 30.1°, θ_min_ = 2.2°
Absorption correction: multi-scan (*SADABS*; Bruker, 1999)	*h* = −9→8
*T*_min_ = 0.974, *T*_max_ = 0.983	*k* = −15→13
13491 measured reflections	*l* = −23→23

### Refinement

**Table:**

Refinement on *F*^2^	Primary atom site location: structure-invariant direct methods
Least-squares matrix: full	Secondary atom site location: difference Fourier map
*R*[*F*^2^ > 2σ(*F*^2^)] = 0.050	Hydrogen site location: inferred from neighbouring sites
*wR*(*F*^2^) = 0.172	H-atom parameters constrained
*S* = 1.06	*w* = 1/[σ^2^(*F*_o_^2^) + (0.0836*P*)^2^ + 0.1125*P*] where *P* = (*F*_o_^2^ + 2*F*_c_^2^)/3
3512 reflections	(Δ/σ)_max_ = 0.001
163 parameters	Δρ_max_ = 0.26 e Å^−^^3^
0 restraints	Δρ_min_ = −0.20 e Å^−^^3^

### Special details

**Table:**

Geometry. Bond distances, angles *etc*. have been calculated using the rounded fractional coordinates. All su's are estimated from the variances of the (full) variance-covariance matrix. The cell e.s.d.'s are taken into account in the estimation of distances, angles and torsion angles
Refinement. Refinement of *F*^2^ against ALL reflections. The weighted *R*-factor *wR* and goodness of fit *S* are based on *F*^2^, conventional *R*-factors *R* are based on *F*, with *F* set to zero for negative *F*^2^. The threshold expression of *F*^2^ > σ(*F*^2^) is used only for calculating *R*-factors(gt) *etc*. and is not relevant to the choice of reflections for refinement. *R*-factors based on *F*^2^ are statistically about twice as large as those based on *F*, and *R*- factors based on ALL data will be even larger.

### Fractional atomic coordinates and isotropic or equivalent isotropic displacement parameters (Å^2^)

**Table:**

	*x*	*y*	*z*	*U*_iso_*/*U*_eq_	
O1	0.28937 (17)	0.48425 (11)	0.21034 (8)	0.0760 (5)	
O2	0.3491 (2)	0.63946 (12)	0.29866 (9)	0.0979 (6)	
N1	0.4211 (2)	0.58198 (13)	0.24703 (9)	0.0682 (5)	
C1	0.5941 (2)	0.58880 (13)	0.21630 (9)	0.0532 (5)	
C2	0.7187 (2)	0.46425 (12)	0.10447 (8)	0.0508 (4)	
C3	0.6602 (3)	0.37116 (13)	0.05213 (9)	0.0577 (5)	
C4	0.4707 (3)	0.31228 (14)	0.05425 (11)	0.0688 (6)	
C5	0.3391 (3)	0.34234 (15)	0.10596 (12)	0.0728 (6)	
C6	0.3994 (2)	0.43709 (14)	0.15601 (10)	0.0590 (5)	
C7	0.5868 (2)	0.49807 (12)	0.15712 (9)	0.0485 (4)	
C8	0.7549 (2)	0.68132 (13)	0.24347 (9)	0.0574 (5)	
C9	0.7396 (2)	0.78613 (12)	0.18692 (8)	0.0492 (4)	
C10	0.5636 (2)	0.85898 (13)	0.17589 (9)	0.0578 (5)	
C11	0.5506 (3)	0.95583 (15)	0.12500 (11)	0.0685 (6)	
C12	0.7117 (3)	0.98059 (16)	0.08532 (11)	0.0753 (7)	
C13	0.8872 (3)	0.90974 (17)	0.09595 (11)	0.0747 (7)	
C14	0.9005 (2)	0.81256 (15)	0.14674 (10)	0.0615 (5)	
C15	0.7953 (3)	0.33322 (18)	−0.00672 (11)	0.0800 (7)	
H2	0.84520	0.50400	0.10450	0.0610*	
H4	0.43230	0.24970	0.01870	0.0830*	
H5	0.21500	0.30080	0.10730	0.0870*	
H8A	0.89380	0.64610	0.24820	0.0690*	
H8B	0.73800	0.70920	0.29620	0.0690*	
H10	0.45330	0.84250	0.20300	0.0690*	
H11	0.43170	1.00420	0.11780	0.0820*	
H12	0.70270	1.04570	0.05090	0.0900*	
H13	0.99740	0.92700	0.06900	0.0900*	
H14	1.02000	0.76470	0.15370	0.0740*	
H15A	0.72950	0.26790	−0.03810	0.1200*	
H15B	0.93050	0.30870	0.02160	0.1200*	
H15C	0.81250	0.39870	−0.04150	0.1200*	

### Atomic displacement parameters (Å^2^)

**Table:**

	*U*^11^	*U*^22^	*U*^33^	*U*^12^	*U*^13^	*U*^23^
O1	0.0529 (6)	0.0788 (8)	0.1006 (10)	−0.0032 (6)	0.0250 (6)	0.0178 (7)
O2	0.0986 (10)	0.0965 (10)	0.1127 (11)	0.0202 (8)	0.0581 (9)	−0.0021 (9)
N1	0.0641 (8)	0.0666 (9)	0.0788 (9)	0.0092 (7)	0.0262 (7)	0.0079 (7)
C1	0.0519 (8)	0.0538 (8)	0.0546 (8)	0.0049 (6)	0.0116 (6)	0.0101 (6)
C2	0.0481 (7)	0.0505 (8)	0.0512 (8)	−0.0029 (6)	0.0018 (6)	0.0074 (6)
C3	0.0650 (9)	0.0501 (8)	0.0521 (8)	0.0031 (7)	−0.0065 (7)	0.0055 (6)
C4	0.0748 (11)	0.0507 (9)	0.0710 (11)	−0.0063 (8)	−0.0148 (9)	0.0047 (8)
C5	0.0573 (9)	0.0597 (10)	0.0930 (13)	−0.0181 (8)	−0.0100 (9)	0.0202 (9)
C6	0.0467 (7)	0.0582 (9)	0.0708 (10)	−0.0024 (7)	0.0062 (7)	0.0187 (7)
C7	0.0439 (7)	0.0472 (7)	0.0524 (8)	−0.0022 (6)	0.0025 (6)	0.0115 (6)
C8	0.0628 (8)	0.0557 (8)	0.0516 (8)	0.0011 (7)	0.0040 (6)	−0.0009 (6)
C9	0.0541 (7)	0.0468 (7)	0.0451 (7)	−0.0011 (6)	0.0041 (6)	−0.0098 (6)
C10	0.0552 (8)	0.0563 (9)	0.0607 (9)	0.0023 (7)	0.0065 (7)	−0.0086 (7)
C11	0.0730 (10)	0.0533 (9)	0.0724 (11)	0.0085 (8)	−0.0059 (9)	−0.0047 (8)
C12	0.0993 (14)	0.0570 (10)	0.0650 (11)	−0.0094 (10)	0.0017 (10)	0.0035 (8)
C13	0.0853 (12)	0.0712 (11)	0.0713 (11)	−0.0149 (10)	0.0242 (9)	−0.0008 (9)
C14	0.0574 (8)	0.0609 (9)	0.0676 (10)	0.0022 (7)	0.0149 (7)	−0.0056 (7)
C15	0.0952 (13)	0.0782 (11)	0.0626 (11)	0.0089 (10)	0.0024 (9)	−0.0121 (9)

### Geometric parameters (Å, º)

**Table:**

O1—N1	1.4587 (19)	C11—C12	1.361 (3)
O1—C6	1.363 (2)	C12—C13	1.370 (3)
O2—N1	1.240 (2)	C13—C14	1.382 (3)
N1—C1	1.3133 (19)	C2—H2	0.9300
C1—C7	1.424 (2)	C4—H4	0.9300
C1—C8	1.483 (2)	C5—H5	0.9300
C2—C3	1.380 (2)	C8—H8A	0.9700
C2—C7	1.3883 (19)	C8—H8B	0.9700
C3—C4	1.396 (3)	C10—H10	0.9300
C3—C15	1.496 (3)	C11—H11	0.9300
C4—C5	1.364 (3)	C12—H12	0.9300
C5—C6	1.373 (2)	C13—H13	0.9300
C6—C7	1.3867 (19)	C14—H14	0.9300
C8—C9	1.509 (2)	C15—H15A	0.9600
C9—C10	1.3854 (19)	C15—H15B	0.9600
C9—C14	1.3691 (19)	C15—H15C	0.9600
C10—C11	1.381 (2)		
			
N1—O1—C6	104.25 (11)	C3—C2—H2	120.00
O1—N1—O2	115.44 (12)	C7—C2—H2	120.00
O1—N1—C1	110.34 (13)	C3—C4—H4	118.00
O2—N1—C1	134.23 (15)	C5—C4—H4	118.00
N1—C1—C7	108.11 (13)	C4—C5—H5	122.00
N1—C1—C8	120.98 (13)	C6—C5—H5	122.00
C7—C1—C8	130.90 (12)	C1—C8—H8A	109.00
C3—C2—C7	119.33 (14)	C1—C8—H8B	109.00
C2—C3—C4	119.02 (15)	C9—C8—H8A	109.00
C2—C3—C15	121.18 (16)	C9—C8—H8B	109.00
C4—C3—C15	119.81 (15)	H8A—C8—H8B	108.00
C3—C4—C5	123.05 (16)	C9—C10—H10	120.00
C4—C5—C6	116.53 (16)	C11—C10—H10	120.00
O1—C6—C5	126.33 (14)	C10—C11—H11	120.00
O1—C6—C7	110.76 (13)	C12—C11—H11	120.00
C5—C6—C7	122.91 (15)	C11—C12—H12	120.00
C1—C7—C2	134.32 (13)	C13—C12—H12	120.00
C1—C7—C6	106.53 (12)	C12—C13—H13	120.00
C2—C7—C6	119.13 (13)	C14—C13—H13	120.00
C1—C8—C9	112.55 (12)	C9—C14—H14	120.00
C8—C9—C10	120.48 (12)	C13—C14—H14	120.00
C8—C9—C14	120.84 (12)	C3—C15—H15A	109.00
C10—C9—C14	118.67 (13)	C3—C15—H15B	109.00
C9—C10—C11	120.54 (14)	C3—C15—H15C	110.00
C10—C11—C12	120.02 (17)	H15A—C15—H15B	110.00
C11—C12—C13	120.11 (17)	H15A—C15—H15C	109.00
C12—C13—C14	120.02 (17)	H15B—C15—H15C	110.00
C9—C14—C13	120.65 (14)		
			
C6—O1—N1—O2	179.19 (14)	C2—C3—C4—C5	0.0 (3)
C6—O1—N1—C1	−0.63 (17)	C3—C4—C5—C6	1.5 (3)
N1—O1—C6—C5	−179.86 (16)	C4—C5—C6—C7	−2.1 (3)
N1—O1—C6—C7	0.19 (16)	C4—C5—C6—O1	177.92 (16)
O1—N1—C1—C7	0.80 (17)	O1—C6—C7—C1	0.26 (17)
O2—N1—C1—C8	0.1 (3)	O1—C6—C7—C2	−178.71 (13)
O2—N1—C1—C7	−178.98 (18)	C5—C6—C7—C1	−179.69 (16)
O1—N1—C1—C8	179.89 (12)	C5—C6—C7—C2	1.3 (2)
C8—C1—C7—C2	−0.9 (3)	C1—C8—C9—C10	65.02 (17)
C8—C1—C7—C6	−179.63 (15)	C1—C8—C9—C14	−116.26 (15)
N1—C1—C8—C9	−99.68 (17)	C8—C9—C10—C11	179.13 (14)
C7—C1—C8—C9	79.18 (19)	C14—C9—C10—C11	0.4 (2)
N1—C1—C7—C6	−0.66 (17)	C8—C9—C14—C13	−178.99 (15)
N1—C1—C7—C2	178.08 (16)	C10—C9—C14—C13	−0.2 (2)
C7—C2—C3—C15	178.79 (14)	C9—C10—C11—C12	−0.2 (3)
C7—C2—C3—C4	−0.8 (2)	C10—C11—C12—C13	−0.2 (3)
C3—C2—C7—C6	0.2 (2)	C11—C12—C13—C14	0.3 (3)
C3—C2—C7—C1	−178.40 (16)	C12—C13—C14—C9	−0.1 (3)
C15—C3—C4—C5	−179.66 (17)		

### Hydrogen-bond geometry (Å, º)

Cg2 is the centroid of the C2–C7 ring.

**Table:**

*D*—H···*A*	*D*—H	H···*A*	*D*···*A*	*D*—H···*A*
C5—H5···O2^i^	0.93	2.49	3.154 (2)	128
C8—H8*B*···*Cg*2^ii^	0.97	3.00	3.6800 (16)	129

Symmetry codes: (i) −*x*+1/2, *y*−1/2, −*z*+1/2; (ii) −*x*+3/2, *y*+1/2, −*z*+1/2.
